# A Comparative Study of Electron Radiation Responses of Pu_2_Zr_2_O_7_ and La_2_Zr_2_O_7_: An *ab* *initio* Molecular Dynamics Study

**DOI:** 10.3390/ma14061516

**Published:** 2021-03-19

**Authors:** Shounuo Zhang, Menglu Li, Haiyan Xiao, Zijiang Liu, Xiaotao Zu

**Affiliations:** 1 School of Physics, University of Electronic Science and Technology of China, Chengdu 610054, China; 201821120122@std.uestc.edu.cn (S.Z.); 202011120518@std.uestc.edu.cn (M.L.); xtzu@uestc.edu.cn (X.Z.); 2 Department of Physics, Lanzhou City University, Lanzhou 730070, China

**Keywords:** pyrochlores, electron radiation, structural amorphization, *ab initio* molecular dynamics simulations

## Abstract

In this study, the response of Pu_2_Zr_2_O_7_ and La_2_Zr_2_O_7_ to electronic radiation is simulated, employing an *ab initio* molecular dynamics method. It is shown that Pu_2_Zr_2_O_7_ undergoes a crystalline-to-amorphous structural transition with 0.3% electronic excitation, while for La_2_Zr_2_O_7_, the structural amorphization occurs with 1.2% electronic excitation. During the microstructural evolution, the anion disorder further drives cation disorder and eventually results in the structural amorphization of Pu_2_Zr_2_O_7_ and La_2_Zr_2_O_7_. The difference in responses to electron radiation between Pu_2_Zr_2_O_7_ and La_2_Zr_2_O_7_ mainly results from the strong correlation effects between Pu 5*f* electrons and the smaller band gap of Pu_2_Zr_2_O_7_. These results suggest that Pu_2_Zr_2_O_7_ is less resistant to amorphization under local ionization rates that produce a low level of electronic excitation, since the level of the concentration of excited electrons is relatively low in Pu_2_Zr_2_O_7_. The presented results will advance the understanding of the radiation damage effects of zirconate pyrochlores.

## 1. Introduction

With the growing demand for nuclear power, the problem of how to treat nuclear waste safely, especially long-lived transuranic (TRU) elements such as plutonium (Pu) and minor actinides (Np, Am) that are generated through spent fuel, has become extremely important [1,2,3]. Pyrochlore-structured oxides with the general formula A_2_B_2_O_7_ (A = Y or another rare earth element; B = Ti, Zr, Sn, or Hf) [4] exhibit a wide range of physical, chemical, and electrical properties, including high ionic conductivity, superconductivity, luminescence, and ferromagnetism [3]. A_2_B_2_O_7_ pyrochlores, thus, are taken as attractive candidates for a variety of applications, including hosts for oxidation catalysts, solid electrolytes, oxygen gas sensors, as well as ceramic thermal barrier coatings [5,6,7]. Particularly great efforts have been devoted to evaluating the potential of pyrochlores as host matrices for immobilization of TRU elements [4,8,9].

Zirconate pyrochlores possess high thermal stability, high chemical durability, and remarkable resistance to radiation-induced amorphization, therefore being of special interest [10,11,12,13]. Wang et al. reported that Gd_2_(Zr_x_Ti_1−x_)_2_O_7_ systems (x = 0, 0.25, 0.5, 0.75, 1) become increasingly radiation resistant with increasing zirconium content under 1 MeV Kr^+^ irradiation [14]. Lian et al. found that among a series of A_2_Zr_2_O_7_ pyrochlores (A = La, Nd, Sm, and Gd), only La_2_Zr_2_O_7_ can be amorphized under ion-beam irradiation [15]. Sickafus and coworkers studied the irradiation response of Er_2_Zr_2_O_7_ at a dose as high as 140 dpa by 350 KeV Xe^+^ at room temperature and found that Er_2_Zr_2_O_7_ cannot be amorphized [16]. Therefore, zirconate pyrochlores would be excellent candidate host matrices for the immobilization of plutonium (Pu) and minor actinides.

In the literature [17,18,19,20,21,22], atomic collision, electronic excitation, and ionization arising from the electronic energy loss of energetic ions have been used to explain the mechanisms of irradiation-induced amorphization. Sattonnay et al. investigated how the composition affects the behavior of pyrochlores under swift heavy ions irradiation and proposed that the susceptibility to amorphization by high electronic excitation is proportional to the cation radii ratio r_A_/r_B_ [23]. Theoretically, the influence of low electronic excitation on microstructural evolution in titanate pyrochlores was explored by Xiao et al., who, using an *ab initio* molecular dynamics (AIMD) method, predicted that structural amorphization occurs under 2% electronic excitation at room temperature [24]. Sassi et al. investigated the interplay between electronic excitation, structure, and composition in lanthanum-based ceramics employing a similar method. They found that when monoclinic-layered perovskite La_2_Ti_2_O_7_ is exposed to a lower degree of electronic excitation, the amorphous transition occurs, whereas a similar phenomenon does not occur in cubic pyrochlore La_2_Zr_2_O_7_ [25]. Furthermore, their results show that La_2_Zr_2_O_7_ can be amorphized at 200 K under 1.6% electronic excitation. These studies demonstrate that electronic excitation may have substantial effects on the microstructural evolution and physical properties of materials.

Thus far, it is not clear how Pu_2_Zr_2_O_7_ pyrochlore, which is a product for immobilization of Pu in zirconate pyrochlores [26,27,28,29], responds to electronic excitation. In this study, a comparative study of the responses of Pu_2_Zr_2_O_7_ and La_2_Zr_2_O_7_ to electronic excitation is made to explore the behaviors of Pu_2_Zr_2_O_7_ under electronic radiation. It is noted that discrepancies exist in microstructural evolution under electronic excitation between Pu_2_Zr_2_O_7_ and La_2_Zr_2_O_7_. The possible reasons have also been explored. The presented results thus gain fundamental insights into the radiation damage effects of Pu_2_Zr_2_O_7_ and may promote related experimental investigations.

## 2. Computational Details

Our calculations are carried out by the *ab initio* molecular dynamics (AIMD) method, as implemented in the Vienna Ab Initio Simulation Package (It was developed by the University of Vienna) (VASP) code [30,31]. In order to describe the exchange-correlation effects between electrons, the generalized gradient approximation (GGA) as parametrized by Perdew and Wang is used [32]. Because AIMD simulation is computationally very expensive, a 1 × 1 × 1 Monkhorst-Pack grid was generally employed in the AIMD simulation [24,33,34]. Hence, a 1 × 1 × 1 Monkhorst-Pack grid is employed in this study as a compromise between computational efficiency and computational accuracy. Computations are performed with a cutoff energy of 300 eV for the plane wave basis set. In our calculations, we employ a 2 × 2 × 2 supercell containing 88 atoms. The Hubbard U correction [35] is considered to modify the strongly correlated Pu 5*f* electrons, and a U_eff_ value of 4 eV is employed [36].

To study the effect of electronic excitation, we remove several electrons from high-lying valence band states. A jellium background is used to compensate for the loss of charge due to electron removal. Within this approximation, one assumes that electrons move in the presence of a neutralizing background consisting of uniformly spread positive charge [37]. After the system reaches equilibrium states, the removed electrons are placed back to mimic the recombination of electrons and holes. This method has made it possible to simulate the role of electronic excitation and has been applied to simulate the structural amorphization of Ge–Sb–Te alloys [38] and pyrochlores [24,25]. For La_2_Zr_2_O_7_ and Pu_2_Zr_2_O_7_, the considered electronic excitation concentrations are 0.3%, 0.6%, and 1.2%. Here, the percentage of electronic excitation concentration corresponds to the number of excited valence electrons to the number of total electrons. The intensity of the e–h pairs that are generated can be estimated by *N*_e-h_ = $\frac{\left(1-R\right)\;\times \;\alpha_{eff\;\times \;F}}{\hbar \omega_{0}}$ [39], where *F* and $\omega_{0}\;$ are the laser fluence and frequency, and *R* and $\alpha_{eff}$ are the reflectivity and effective absorption coefficient for the sample [40,41,42,43]. Under laser beam irradiation, the laser fluence at 400 nm for 1% excitation in La_2_Zr_2_O_7_ is about 8.6 × 10^2^–4.9 × 10^3^ mJ/cm^2^. The AIMD simulation is conducted employing an isothermal–isochoric ensemble, and the temperature is controlled by the Nosé–Hoover thermostat. The simulation time is 6 ps, and the time step is 3 fs.

## 3. Results and Discussions

### 3.1. Ground State Properties of Pu_2_Zr_2_O_7_ and La_2_Zr_2_O_7_

**Figure 1:**
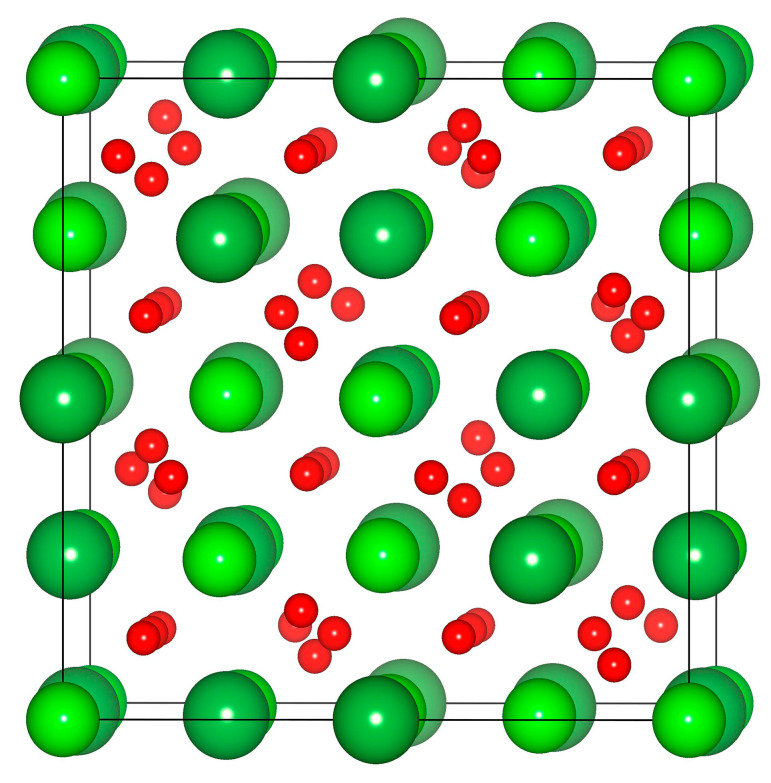
Schematic view of the geometrical structures of La_2_Zr_2_O_7_. The dark green, yellow-green, and red spheres represent La, Zr, and O atoms, respectively.

Structural optimization is first performed on Pu_2_Zr_2_O_7_ and La_2_Zr_2_O_7_. The Schematic view of the geometrical structures of La_2_Zr_2_O_7_ is shown in Figure 1. The calculated lattice constants, oxygen positional parameter *x_O8f_*, as well as bonding distances for Pu_2_Zr_2_O_7_ and La_2_Zr_2_O_7_, are summarized in Table 1, along with the available theoretical and experimental results for comparison. For Pu_2_Zr_2_O_7_, the obtained lattice constant of 10.802 Å is slightly larger than the experimental value of 10.70 Å [44], whereas it is consistent with the theoretical result of 10.802 Å [36]. The lattice constant of La_2_Zr_2_O_7_ is determined to be 10.879 Å, which is in reasonable agreement with the experimental value of 10.805 Å [45] and comparable to the calculated value of 10.696 Å [2]. The relatively larger lattice constant for La2Zr2O7 is mainly due to its larger ionic radius, i.e., ~1.16 Å for La^3+^ and ~1.1 Å for Pu^3+^ [46]. With regard to oxygen positional parameter *x_O48f_*, the calculated value of 0.335 for Pu_2_Zr_2_O_7_ is the same as other calculations of 0.335 [36]. For La_2_Zr_2_O_7_, the calculated value of 0.333 agrees well with the experimental and other calculated values [2,45]. Generally, the pyrochlores with the *x_O48f_* value being closer to 0.375 are more resistant to structural amorphization under ion irradiation [26,47]. It is noted that the *x_O48f_* value for Pu_2_Zr_2_O_7_ is slightly larger than that of La_2_Zr_2_O_7_, suggesting that Pu_2_Zr_2_O_7_ and La_2_Zr_2_O_7_ may have different responses to ion irradiation.

### 3.2. Microstructural Evolution in La_2_Zr_2_O_7_ under Electronic Excitation

**Figure 2:**
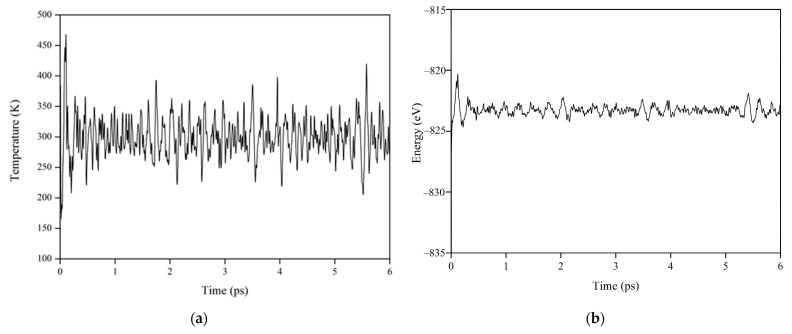
Variation of (**a**) temperature and (**b**) total energy with time for La_2_Zr_2_O_7_ with 0.3% electron excitation.

In order to explore the response of La_2_Zr_2_O_7_ to electronic radiation, AIMD simulations are first carried out with an electronic excitation concentration of 0.3%. Figure 2 shows a variation of temperature and total energy with time for La_2_Zr_2_O_7_ with 0.3% electronic excitation at 300 K. It is obvious that the simulation time of 6 ps is long enough so that the system can reach equilibrium states.

**Figure 3:**
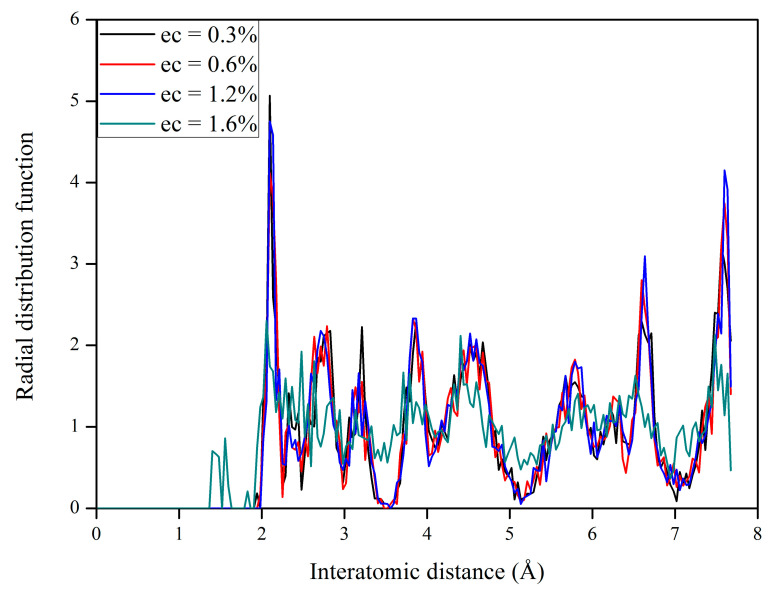
Radial distribution function (RDF) for La_2_Zr_2_O_7_ with 0.3%, 0.6%, 1.2%, and 1.6% electron excitations.

**Figure 4:**
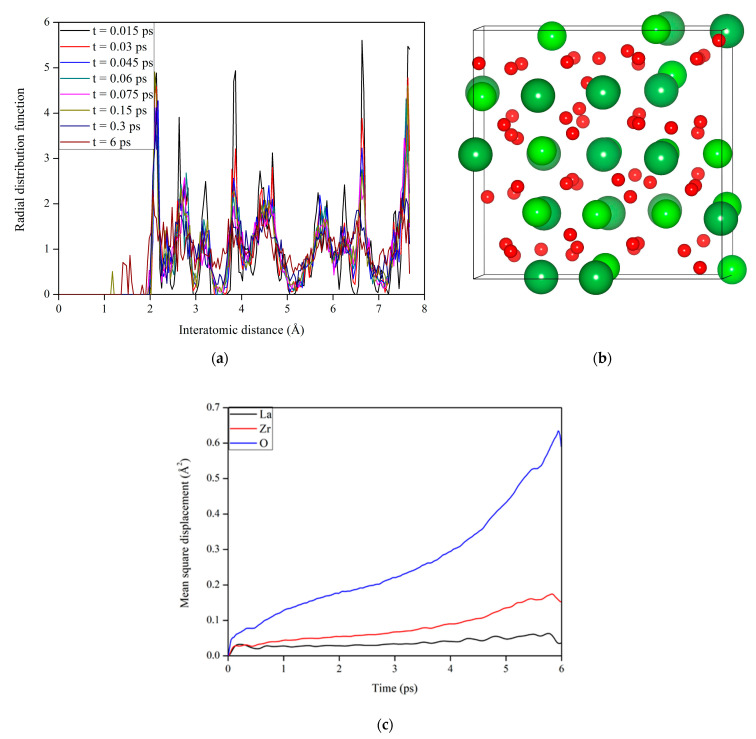
(**a**) Variation of radial distribution function (RDF) with time, (**b**) schematic view of the geometrical structure, and (**c**) variation of mean square displacement (MSD) with time for La_2_Zr_2_O_7_ with 1.6% electron excitation.

To investigate how the electronic excitation concentration affects microstructural evolution in La_2_Zr_2_O_7_ at 300 K, electronic excitation concentration of 0.3%, 0.6%, 1.2%, and 1.6% are considered. Based on each equilibrium state, the radial distribution function (RDF) analysis is then carried out. Figure 3 shows the RDF for La_2_Zr_2_O_7_ with 0.3%, 0.6%, 1.2%, and 1.6% electronic excitations. For electronic excitations of 0.3%, 0.6%, and 1.2%, it is noted that the structure is ordered at both short-range and long-range distances, meaning that La_2_Zr_2_O_7_ still remains a pyrochlore structure. Here, the short-range correlation means the bonding interaction, and the long-range correlation corresponds to a nonbonding interaction. In the case of 1.6% electronic excitation, the structure retains a short-range order but has lost its long-range order, suggesting that 1.6% electronic excitation can induce a crystalline-to-amorphous transition in La_2_Zr_2_O_7_ at room temperature. In the literature, a similar phenomenon was observed by Sassi et al. [25]. Variation of RDF with time for La_2_Zr_2_O_7_ with 1.6% electronic excitation is displayed in Figure 4a. It is shown that the structural amorphization starts at t = 0.075 ps and the structure is completely amorphized at t = 0.3 ps, i.e., under 1.6% electronic excitation the crystalline-to-amorphous transition occurs very fast.

Figure 4b shows a schematic view of the geometrical structure for La_2_Zr_2_O_7_ with 1.6% electronic excitation. Compared with the pyrochlore structure presented in Figure 1, it can be seen that the structure is disordered after 1.6% electronic excitation. Furthermore, the degree of anion disorder is much larger than that of cation disorder. We also explore the variation of mean square displacement (MSD) with time for La_2_Zr_2_O_7_ with 1.6% electronic excitation, and the results are presented in Figure 4c. It is found that the mean square displacement of oxygen is considerably larger than that of La and Zr. These results indicate that the displacement of oxygen drives the pyrochlore of La_2_Zr_2_O_7_ to undergo a crystalline-to-amorphous transition under 1.6% electronic excitation. Theoretically, Xiao et al. also suggested that the amorphization of titanate pyrochlores is mainly contributed by the displacement of oxygens [24]. Experimentally, Lian et al. found that under ion irradiation, anion disorder precedes cation disorder in Gd_2_Ti_2_O_7_, Er_2_Ti_2_O_7_, and La_2_Ti_2_O_7_ [48]. In this study, it is noted that the MSD increases with the increasing time rather than vibrates slightly after the system reaches equilibrium states. A similar phenomenon has been found in the literature [49], where the La/Zr/O atoms in the amorphous structure also diffuse rather than vibrate at their equilibrium sites.

### 3.3. Microstructural Evolution in Pu_2_Zr_2_O_7_ under Electronic Excitation

**Figure 5:**
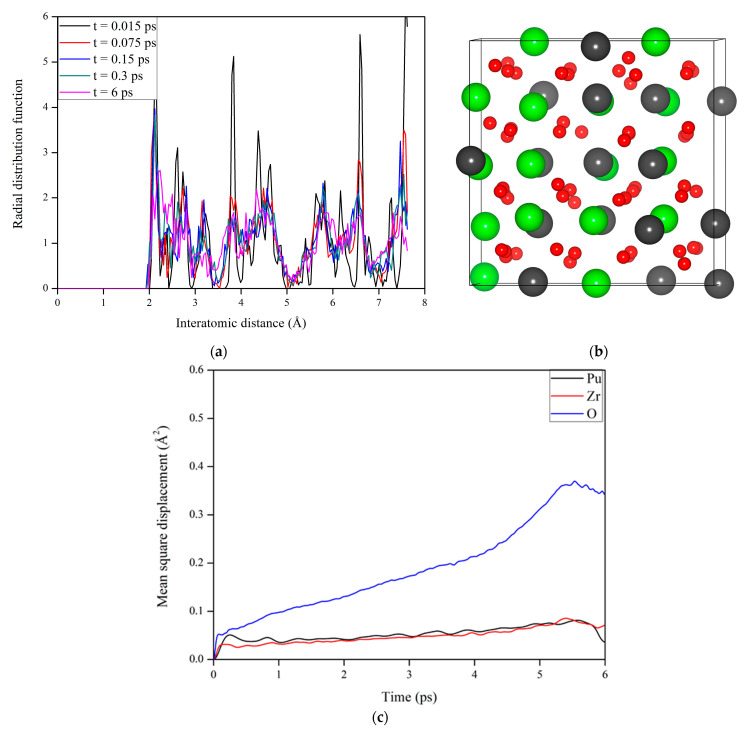
(**a**) Variation of radial distribution function (RDF) with time, (**b**) schematic view of the geometrical structure, and (**c**) variation of mean square displacement (MSD) with time for Pu_2_Zr_2_O_7_ with 0.3% electron excitation.

To explore how the Pu_2_Zr_2_O_7_ pyrochlore responds to electronic excitation, AIMD simulation is also carried out on Pu_2_Zr_2_O_7_, in which 0.3% electrons are excited at 300 K. The corresponding variation of RDF with time for Pu_2_Zr_2_O_7_ with 0.3% electronic excitation is illustrated in Figure 5a. We found that at t = 0.3 ps the structure becomes disordered at a long-range distance, and with time evolution, the structure is eventually completely amorphized. Compared with the case of La_2_Zr_2_O_7_, the crystalline-to-amorphous transition occurs more easily in Pu_2_Zr_2_O_7_, since the threshold electronic concentration of 0.3% is much lower than that of 1.6% for La_2_Zr_2_O_7_. These results suggest that the Pu_2_Zr_2_O_7_ should be readily amorphized under local ionization rates that produce a low level of electronic excitation. Theoretically, Shen et al. suggested that the influences of different types of point defects on the thermomechanical properties of Pu_2_Zr_2_O_7_ and Gd_2_Zr_2_O_7_ show somewhat different character, and Pu_2_Zr_2_O_7_ has been suggested to be more susceptible to radiation-induced amorphization than other zirconate pyrochlores like Gd_2_Zr_2_O_7_ [50].

To explore the origin of the structural amorphization induced by electronic excitation in Pu_2_Zr_2_O_7_, we plot the variation of mean square displacement with time for Pu_2_Zr_2_O_7_ with 0.3% electron excitation in Figure 5c. It is shown that the mean square displacement of anions is considerably larger than that of cations. Figure 5b also shows that the disorder of anions is more significant than that of cations. These results suggest that the structural amorphization is also driven by anion disordering, similar to the case of La_2_Zr_2_O_7_ discussed above and the cases of titanate pyrochlores reported by Xiao et al. [24].

### 3.4. Origin of the Different Electronic Radiation Responses between La_2_Zr_2_O_7_ and Pu_2_Zr_2_O_7_

In order to explain the different electronic radiation responses between Pu_2_Zr_2_O_7_ and La_2_Zr_2_O_7_, we further analyze their geometrical and electronic structures. Comparing the bonding distances in Pu_2_Zr_2_O_7_ and La_2_Zr_2_O_7_ (see Table 1), we find that the values of ~2.587 Å for <Pu-O*_48f_*>, ~2.317 Å for <Pu-O_8b_>m and ~2.099 Å for <Zr-O_48f_> are slightly smaller than the values of ~2.635 Å for <La-O_48f_>, ~2.339 Å for <La-O_8b_>, and 2.106 Å for <Zr-O_48f_>, respectively. These results mean that stronger bonding interactions exist in Pu_2_Zr_2_O_7_ than in La_2_Zr_2_O_7_. However, the band gap of 2.12 eV for Pu_2_Zr_2_O_7_ is smaller than the value of 3.52 eV for La_2_Zr_2_O_7_, i.e., the valence electrons in Pu_2_Zr_2_O_7_ are more easily to be excited to the conduction bands if enough energy is provided.

**Figure 6:**
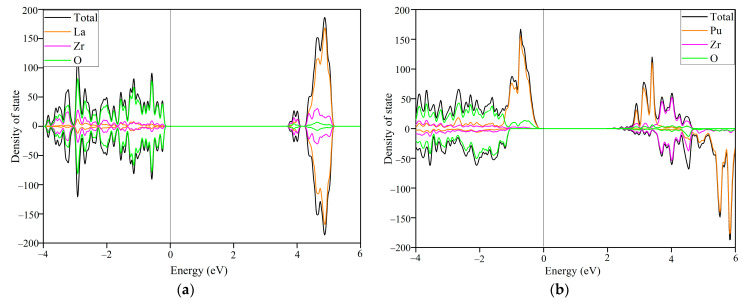
A comparison of the atomic projected density of state (DOS) distribution for ideal (**a**) La_2_Zr_2_O_7_ and (**b**) Pu_2_Zr_2_O_7_.

Figure 6 presents the total and projected density of state (DOS) distributions for idealLa_2_Zr_2_O_7_ and Pu_2_Zr_2_O_7_. For La_2_Zr_2_O_7_ (see Figure 6a), O 2*p* orbital dominates and hybridizes with very few La 5*d* and Zr 4*d* orbitals at the valence band maximum (VBM), and few Zr 4*d* and O 2*p* orbitals contribute to the conduction band minimum (CBM). For Pu_2_Zr_2_O_7_ (see Figure 6b), it is shown that the Pu 5*f* orbital dominates and hybridizes with very few O 2*p* and Zr 4*d* orbitals at the VBM, and the CBM are contributed by Pu 5*f* orbital and very few Zr 4*d* and O 2*p* orbitals. On the one hand, because of the strong correlation effects between Pu 5*f* electrons, the O 2*p* electrons in Pu_2_Zr_2_O_7_ are more readily to be excited than Pu 5*f* electrons. On the other hand, in spite of the stronger <Pu-O> bonding interaction in Pu_2_Zr_2_O_7_ than the <Zr-O> bonding interaction in La_2_Zr_2_O_7_ at valence bands, the O 2*p* electrons in Pu_2_Zr_2_O_7_ are more easily to be excited to the conduction bands due to the much smaller band gap. Consequently, anion disorder drives cation disorder and eventually results in structural amorphization.

## 4. Conclusions

In summary, the microstructural evolution in Pu_2_Zr_2_O_7_ and La_2_Zr_2_O_7_ under electron radiation has been investigated by *ab initio* molecular dynamics simulations. It is shown that Pu_2_Zr_2_O_7_ is more susceptible to electron radiation than La_2_Zr_2_O_7_, since the crystalline-to-amorphous structural transition at 300 K occurs at 0.3% electronic excitation for Pu_2_Zr_2_O_7_ and 1.6% for La_2_Zr_2_O_7_. In both compounds, the degree of anion disorder is much larger than the degree of cation, i.e., the structural amorphization is driven by anion disorder. In Pu_2_Zr_2_O_7_, there are strong correlation effects between Pu 5*f* electrons, resulting in O 2*p* electrons being more readily excited. Furthermore, the band gap of Pu_2_Zr_2_O_7_ is much smaller than that of La_2_Zr_2_O_7_. Consequently, the O 2*p* electrons in Pu_2_Zr_2_O_7_ are more easily to be excited to the conduction bands, and Pu_2_Zr_2_O_7_ is less resistant to electron radiation than La_2_Zr_2_O_7_.

## Figures and Tables

**Table 1:** The calculated lattice constants a_0_ (Å), O_48f_ position parameter *x* (*x_O48f_*), and bonding distances (Å) for La_2_Zr_2_O_7_ and Pu_2_Zr_2_O_7_. E_g_ represents the band gap; d<A-B>: bonding distances between A and B atoms (A = La, Pu, or Zr; B = O_48f_ or O_8b_).

Compounds	a_0_	*x_O48f_*	Eg (eV)	d<La-O_48f_ >	d<La-O_8b_ >	d<Pu-O_48f_>	d<Pu-O_8b_>	d<Zr-O_48f_>
La_2_Zr_2_O_7_	10.879	0.333	3.58	2.635	2.339	–	–	2.106
Cal. [2]	10.696	0.3346	3.52	2.589	2.316	–	–	2.096
Exp. [45]	10.805	0.332	–	2.635	2.339	–	–	2.105
Pu_2_Zr_2_O_7_	10.802	0.335	2.12	–	–	2.587	2.317	2.099
Cal. [36]	10.802	0.335	2.37	–	–	2.615	2.339	2.117
Exp. [44]	10.70	–	–	–	–	–	–	–

## Data Availability

The raw/processed data required to reproduce these findings cannot be shared at this time as the data also form part of an ongoing study.
